# Identification and characterization of short tandem repeats in the Tibetan macaque genome based on resequencing data

**DOI:** 10.24272/j.issn.2095-8137.2018.047

**Published:** 2018-05-12

**Authors:** San-Xu Liu, Wei Hou, Xue-Yan Zhang, Chang-Jun Peng, Bi-Song Yue, Zhen-Xin Fan, Jing Li

**Affiliations:** 1 Key Laboratory of Bio-Resource and Eco-Environment of Ministry of Education, College of Life Sciences, Sichuan University, Chengdu Sichuan 610065, China; 2 Sichuan Key Laboratory of Conservation Biology on Endangered Wildlife, College of Life Sciences, Sichuan University, Chengdu Sichuan 610065, China

**Keywords:** Tibetan macaque (*Macaca thibetana*) genome, Short tandem repeats, Variation analysis, Polymorphism, Next-generation sequencing

## Abstract

The Tibetan macaque, which is endemic to China, is currently listed as a Near Endangered primate species by the International Union for Conservation of Nature (IUCN)(2017). Short tandem repeats (STRs) refer to repetitive elements of genome sequence that range in length from 1–6 bp. They are found in many organisms and are widely applied in population genetic studies. To clarify the distribution characteristics of genome-wide STRs and understand their variation among Tibetan macaques, we conducted a genome-wide survey of STRs with next-generation sequencing of five macaque samples. A total of 1 077 790 perfect STRs were mined from our assembly, with an N50 of 4 966 bp. Mono-nucleotide repeats were the most abundant, followed by tetra- and di-nucleotide repeats. Analysis of GC content and repeats showed consistent results with other macaques. Furthermore, using STR analysis software (lobSTR), we found that the proportion of base pair deletions in the STRs was greater than that of insertions in the five Tibetan macaque individuals (*P*<0.05, *t*-test). We also found a greater number of homozygous STRs than heterozygous STRs (*P*<0.05, *t*-test), with the Emei and Jianyang Tibetan macaques showing more heterozygous loci than Huangshan Tibetan macaques. The proportion of insertions and mean variation of alleles in the Emei and Jianyang individuals were slightly higher than those in the Huangshan individuals, thus revealing differences in STR allele size between the two populations. The polymorphic STR loci identified based on the reference genome showed good amplification efficiency and could be used to study population genetics in Tibetan macaques. The neighbor-joining tree classified the five macaques into two different branches according to their geographical origin, indicating high genetic differentiation between the Huangshan and Sichuan populations. We elucidated the distribution characteristics of STRs in the Tibetan macaque genome and provided an effective method for screening polymorphic STRs. Our results also lay a foundation for future genetic variation studies of macaques.

## INTRODUCTION

Short tandem repeats (STRs), also known as microsatellites, are highly variable repetitive elements with nucleotide motifs of 1–6 bp and are ubiquitous in most eukaryotic organisms (Oliveira et al., 2006). Microsatellite mutations are generally derived from replication slippage, leading to the insertion or deletion of one or several repeat motifs (Levinson & Gutman, 1987; Schlötterer, 2000; Schlötterer & Tautz, 1992). STRs located in both the coding and non-coding areas of DNA can affect gene expression and transcription (Chistiakov et al., 2006; Li et al., 2004), especially STRs in the coding regions. For example, certain human neurological disorders are caused by trinucleotide repeat expansions in coding regions (La Spada et al., 1991; MacDonald et al., 1993). Previous studies have also indicated that the mutation processes of STR loci can vary, even among closely related species, resulting in interspecific differences in STR allele size (Rubinsztein et al., 1995; Schlötterer, 1998; Webster et al., 2002). STRs are frequently applied to genetic mapping, forensic identification, and phylogenetic studies as molecular markers due to their high abundance, neutrality, high variability, and codominance (Schlötterer, 1998). Recent studies have also emphasized the role of STRs in the genetic architecture of quantitative human traits (Gymrek et al., 2016). In addition, STRs have been used as molecular markers for the study of genetic diversity and population genetic structure (Qin et al., 2016; Sunnucks, 2000). Here, we determined genome-wide STR variation in Tibetan macaque populations based on high-throughput sequencing.

The Tibetan macaque (*Macaca thibetana*) is one of 23 extant species of the genus *Macaca* (Primates: Cercopithecidae) and is endemic to China. Its geographic distribution extends from southwest to east regions in China (Jiang et al., 1996). Jiang et al. (1996) divided *M. thibetana* into four subspecies according to variations in external morphology, hair color, cranial features, and geographical distribution: namely, *Macaca thibetana thibetana*, *Macaca thibetana pullus*, *Macaca thibetana huangshanensis*, and *Macaca thibetana guizhouensis*. The species is an increasingly studied animal model in biomedical research due to its ease of domestication, large body size, long life span, and physiological characteristics analogous to those of humans (Yi et al., 2012). Studies on intraocular pressure (Liu et al., 2011) and liver transplantation (Ji et al., 2015) are two good application examples. However, habitat destruction, deforestation, and illegal poaching have led to considerable fragmentation of natural *Macaca* populations, with a resulting decrease in wild Tibetan macaque populations observed in recent years (Jiang et al., 2016). Currently, the Tibetan macaque is a vulnerable and endangered species (Jiang et al., 2016), and is classified as a Category II species under the Chinese Wild Animal Protection Law. Thus, studies on its genetic variability and diversity are urgently needed to assess genetic differentiation and develop effective conservation strategies. To date, previous studies have recognized 56 STR markers in Tibetan macaques by genomic library construction and cross-species amplification (Jia et al., 2011, 2012; Li et al., 2014; Yang et al., 2017). Nevertheless, the numbers of identified STR markers are insufficient for accurate estimation, conservation, and management of Tibetan macaques. Most STRs in the Tibetan macaque genome remain unidentified and there is no information available on the comparative analysis of whole genome STRs due to the lack of genomic data.

Whole genome profiles of STR variations in non-model organisms have not yet been conducted, despite the discovery of STRs in the 1980s (Guichoux et al., 2011). This is due to the associated expense as well as the inefficient and laborious construction of genomic libraries and enrichment of clones containing STR motifs (Castagnone-Sereno et al., 2010; Powell et al., 1996). Recently, the advent of high-throughput sequencing technology has produced considerable genomic data, providing the opportunity for genome-wide analysis of STR variations. To date, STR marker development and polymorphism analyses based on high-throughput sequencing data have been performed in humans (Willems et al., 2014), cattle (Xu et al., 2016), maize (Qu & Liu, 2013), pig (Liu et al., 2017a), faba bean (Abuzayed et al., 2017), and *Angelica gigas Nakai* (Gil et al., 2017). However, the distribution and variation of genome-wide STRs in the Tibetan macaque remain largely unknown. To identify and characterize all potential STR loci and clarify the differences in STRs among Tibetan macaque individuals, we systematically profiled STR distribution in the Tibetan macaque genome and STR variations between Emei, Jianyang, and Huangshan individuals based on whole genome resequencing data. These profiling results will contribute to our understanding of Tibetan macaque species variability.

## MATERIALS AND METHODS

### Genomic samples and high-throughput sequencing

Five Tibetan macaques, two obtained from Huangshan Mountain (Anhui Province, HS) and three captured from Emei Mountain (one individual) and Jianyang city (two individuals) in Sichuan Province (SC), China, were used for whole genome resequencing. Genomic DNA was isolated from blood and muscle tissue samples (Supplementary Table S1). All procedures were approved by the local Ethics Review Board and were in accordance with all relevant national and international regulations. We generated 150-bp paired-end reads with insertion sizes of approximately 350 bp on the Illumina HiSeq X Ten platform. In total, 93.68–104.19 Gb of clean data were obtained after filtering out low-quality reads using the NGS QC toolkit with a quality score of 20 and percentage of read length less than 75% of given quality (Patel & Jain, 2012) at a sequencing depth of over 30× in the five samples, respectively. The resequencing genome data were deposited in the GSA dataset (http://bigd.big.ac.cn/gsa/) under accession number CRA000789.

### Genome assembly and characterization of STRs

The high-quality clean reads of one Emei Mountain macaque were assembled using the short reads assembling program ABySS 2.0 (Simpson et al., 2009). The resulting short contigs (<150 bases) were excluded from the assembly. We then used MSDB v2.4 (Du et al., 2013) to search STRs using the same settings and parameters as described previously (Liu et al., 2017b). 

### Genotyping of STRs based on resequencing data

Genotyping of the STRs was performed using lobSTR software (version 4.0.6), a rapid and accurate algorithm for STR profiling based on whole-genome sequencing data (Gymrek et al., 2012). The algorithm can avoid gapped alignment and address specific noise patterns in STR calling. In this study, a lobSTR reference was first constructed based on rhesus macaque (GCF_000772875.2) STR data (generated in Liu et al., 2017b) using lobstr_index.py script. The resequencing data were then aligned to the rhesus macaque reference genome with default parameters. The output BAM files were sorted with SAMtools. Finally, we analyzed STR allelotypes based on the alignment files of the five samples. In total, we obtained 840 356 STRs across the five macaque individuals after removing low-quality STRs (QUAL<30), which were used for downstream analyses. Statistical analyses were performed using SPSS version 19.

### Genetic relatedness analysis of STRs

Tetranucleotide STR loci from all five samples were employed for genetic analysis. Each STR allele different from the reference allele size was coded as a binary string of all zeros, and the remaining alleles were set to one. Genetic distance was calculated using the vegan package in the R program (version 3.0.2) (Team, 2000). Finally, we constructed a phylogenetic tree based on the neighbor-joining method in MEGA 5.2 (Hall, 2013).

### Screening of polymorphic STRs

Allele numbers of each STR loci were counted based on genotyping data, and high-quality, polymorphic tetranucleotide loci were screened using VCFtools by the application of “- -min-alleles 4” and “- -maf 0.1” parameters. Ten loci from these STRs were randomly selected to validate efficiency and polymorphism in the 16 Tibetan macaque samples by agarose gel electrophoresis and capillary electrophoresis. Cervus software was employed to calculate the observed number of alleles (*N*a), mean observed heterozygosity (*H*o), mean expected heterozygosity (*H*e), and polymorphic information content (*PIC*).

## RESULTS

### Sequencing, assembly, and STR overview of the Tibetan macaque genome

We resequenced the genomes of five Tibetan macaques from Sichuan and Huangshan populations using Illumina sequencing technology, generating over 30-fold sequencing depth for each individual. After data filtering, we obtained a total of 312 252 249 clean sequencing reads with 150-bp and 350-bp insert fragments, corresponding to 93.68–95.45 G base pairs with GC content of approximately 45% and over 94.06% Q20 bases (base quality more than 20) in each sample (Supplementary Table S1). In the assembled genome, we acquired a total of 1 223 752 contigs with a total length of 2.66 Gb and N50 of 4 966 bp. The average GC content of the genomic sequences was 40.48%. The maximum and average lengths of the contigs were 97 085 bp and 2 172.38 bp, respectively (Table 1). The length distributions of the contigs can be seen in Supplementary Figure S1.

**Table 1:** Assembly results for the *M. thibetana* genome

Item	Number
Total number of sequences examined (*n*)	1 223 752
Total size of examined sequences (bp)	2 658 459 556
Mean length of examined sequences (bp)	2 172.38
N50	4 966
N90	1 108
GC content (%)	40.48%
Number of sequences containing STRs (*n*)	521 523
Number of sequences containing more than one STR (*n*)	252 041

Hereafter, we analyzed the distribution of STRs in the assembly using MSDB2.4 software. All 1 223 752 contigs generated in this study were used to identify potential STRs with minimum repeats of 12, 7, 5, 4, 4, and 4 for all motifs from mono- to hexa-nucleotide STRs, respectively. As expected, perfect STRs were the most abundant category, followed by compound STRs, interrupted compound and interrupted perfect STRs, and complex STRs (Supplementary Table S2). A total of 1 077 790 perfect STRs (accounting for approximately 0.78% of the assembly) were mined in 521 523 contigs, with 252 041 sequences containing more than one STR (Table 1). The frequency, average length, and GC content of the potential STRs were also analyzed. Among the 1 077 790 STRs, mononucleotide repeats with the highest frequency of 234.7 loci/Mb were the most abundant types, accounting for 57.89% of the total number of STRs, followed by tetra- (181 344 loci, 16.83%) and di-nucleotide (165 769 loci, 15.38%) repeats, with hexanucleotide (6 347 loci, 0.59%) repeats the least abundant (Table 2). Average length of the STR core sequences increased with repeat motifs from mono- to hexa-nucleotide STRs, except for trinucleotide repeats, with the total average length found to be 19.19 bp. Analysis of GC content for the six STR types showed that GC content was highest in dinucleotide STRs, accounting for 48.75% of dinucleotide STRs in the assembly, followed by tri-, hexa- and tetra-nucleotide repeats. The lowest GC-content was found in the mononucleotide STRs, accounting for 0.48% in the genome (Table 2).

**Table 2:** Number, length, frequency, density, and GC content of perfect STRs

Types	Total Counts	Total Length (bp)	Average Length (bp)	Frequency (loci/Mb)	Density (bp/Mb)	GC Length	GC Content (%)
Mono-	623 930	10 397 456	16.66	234.7	3 911.083	50 200	0.48
Di-	165 769	3 646 216	22	62.36	1 371.552	1 777 603	48.75
Tri-	64 529	1 216 338	18.85	24.27	457.535	436 005	35.85
Tetra-	181 344	4 372 964	24.11	68.21	1 644.924	1 407 970	32.20
Penta-	35 871	873 535	24.35	13.49	328.587	230 585	26.40
Hexa-	6 347	172 866	27.24	2.39	65.025	60 271	34.87
Total	1 077 790	20 679 37 (0.78%)	19.19	405.42	7 778.706	3 962 634	19.16

**Figure 1:**
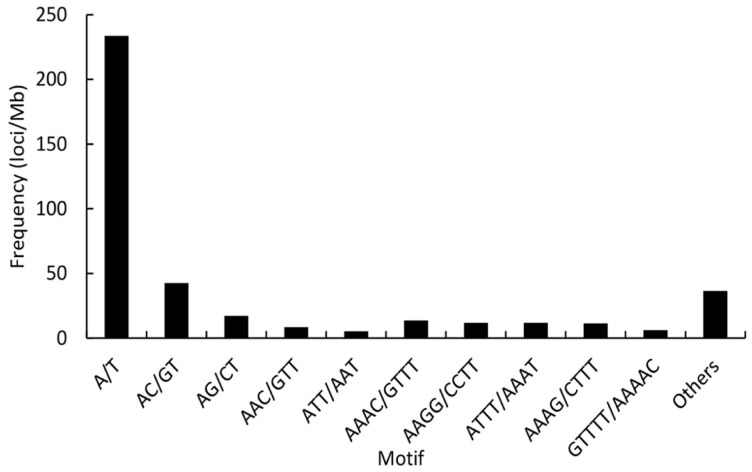
Distribution of STR motifs in *M. thibetana*

**Figure 2:**
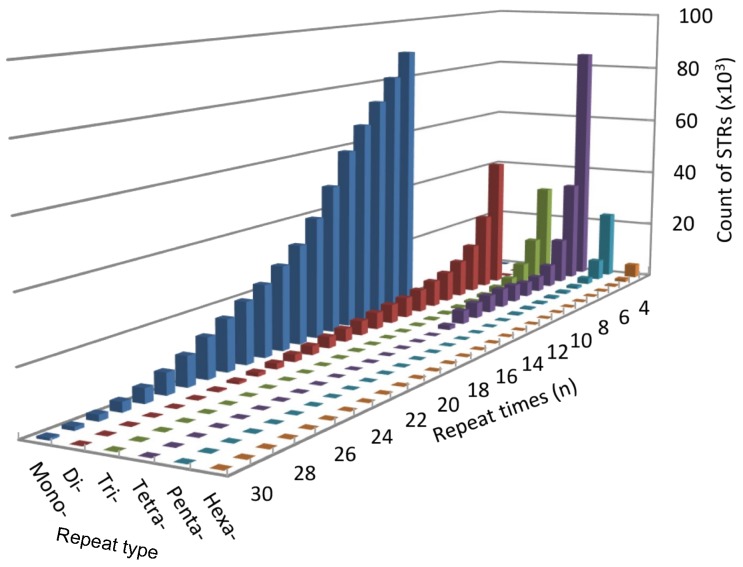
Distribution of STR types in the *M. thibetana* genome by repeat time

We also found that most repeat motifs were AT-rich, except for the dinucleotide repeats. With a frequency of 233.81 loci/Mb, the A/T mononucleotide repeat was the most frequent motif in the STRs, followed by the AC/GT (42.6 loci/Mb), AG/CT (17.09 loci/Mb), AAAC/GTTT (13.53 loci/Mb), ATTT/AAAT (11.76 loci/Mb), AAGG/CCTT (11.7 loci/Mb), AAAG/CTTT (11.35 loci/Mb), AAC/GTT (8.47 loci/Mb), GTTTT/AAAAC (6.22 loci/Mb), and ATT/AAT (5.28 loci/Mb) motifs. The frequency of the remaining motifs was 36.52 loci/Mb (Figure 1). The number of major repeats ranged from 12 to 40 for mono-, 7 to 27 for di-, 5 to 20 for tri-, 4 to 15 for tetra-, 4 to 12 for penta-, and 4 to 8 for hexa-nucleotide repeat, respectively (Figure 2). These loci accounted for 97.89% of the total counts of each STR type.

### Alignment and genotyping of STRs

**Figure 3:**
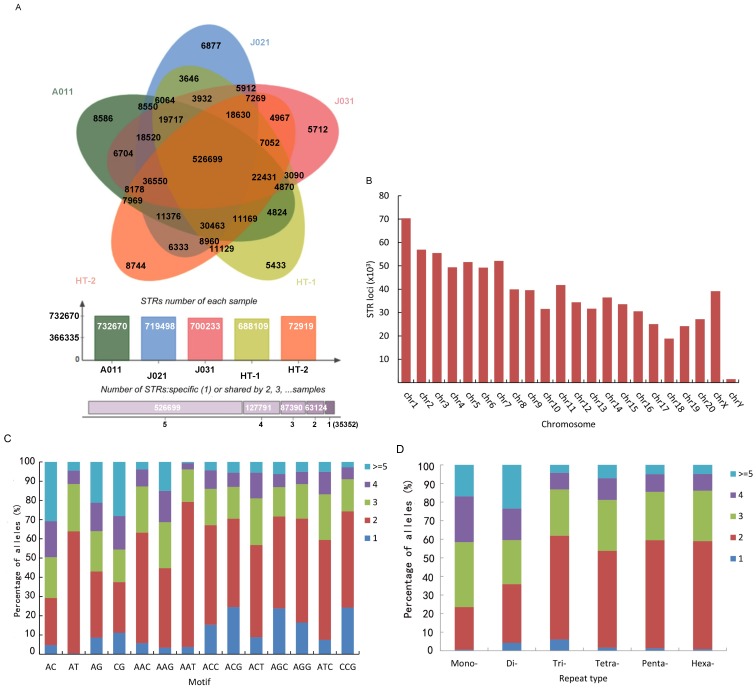
Alignment results of STRs

To explore differences in STR distribution among the Tibetan macaques, we used lobSTR software to examine the high-throughput sequencing data of the five individuals from the Huangshan and Sichuan populations (Supplementary Table S1). A total of 1 060 419 STRs were found, with the 1 294 455 perfect STRs located in the rhesus macaque chromosomes used as a reference. After removing low-quality STRs (QUAL<30), we achieved a total of 840 356 STRs across the five macaques. On average, we obtained genotypes for 713 685.8 STR loci, with a mean coverage of 5.94-fold per individual, of which 354 834.2 STR loci (49.72%) had 5-fold greater call coverage (Figure 3A). Further observation showed that 526 699 STR loci (62.68%) of the 840 356 STRs called in our study were shared by the five macaques and 35 352 STR loci (4.20%) were unique to one individual. In addition, 63 124, 87 390, and 127 791 STRs were identified in two, three, and four individuals, respectively (Figure 3A). The STR loci showed greatest alignment to chr 1, followed by chr 2, chr3, chr 7, and chr 5, and finally chr Y (Figure 3B). Comparison of allele number of di- and tri-nucleotide STR motifs indicated that AT repeats had the highest proportion in dinucleotide STR motifs with greater than one allele, followed by AC, AG, and CG repeats. For trinucleotide STR motifs with more than one allele, AAG, AAT, and AAC were found at higher proportions. Conversely, for STR motifs with only one allele (no polymorphism), the proportions of CG and CCG were higher than those of the other motif types (Figure 3C).

### Screening and initial validation of polymorphic STRs

Analysis of the allele number of STRs among the five individuals indicated that loci with only one allele were underrepresented in all individuals, accounting for 1.41% (11 869 loci) of all loci. Among these loci, trinucleotide STRs had the highest proportion, accounting for 6.03% (1 726 loci) of the total number of trinucleotide STRs; and mononucleotide STRs were relatively underrepresented, accounting for 0.54% (3 205 loci) of mononucleotide STRs. STR loci with two alleles accounted for the highest proportion in di- to hexa-nucleotide STRs. The proportion of STRs with two alleles (22.93%, 31.51%, 55.88%, 52.12%, 58.09%, and 58.15% from mono- to hexa-nucleotide STRs, respectively) increased with motif length. Loci with more than four alleles (pSTRs) had the highest proportion in mono- (245 673 loci, 41.56%) and di-nucleotide loci (52 180 loci, 40.42%), followed by tetra- (13 307 loci, 18.84%), penta- (2 560 loci, 14.50%), hexa- (456 loci, 13.85%), and tri-nucleotide STRs (3 771 loci, 13.17%) (Figure 3D). pSTRs accounted for 37.83% of the total STRs.

A: Number of shared and specific STRs among the five *M. thibetana* samples. B: Distribution of STRs mapped to reference chromosomes. C: Comparison of allele number for di- and tri-nucleotide STR motifs. D: Comparison of allele number for the six types of STRs.

We also characterized 3 325 tetranucleotide STRs that showed at least four alleles with a frequency of over 0.1. The coordinate information of these loci can be seen in Supplementary Table S3. Ten loci from these STRs were randomly selected to validate efficiency and polymorphism. Among them, the forward primers of six STR loci that were amplified successfully in the 16 samples were labeled by fluorescence (FAM/HEX). Finally, four to seven alleles were detected across each locus of the six loci. The *H*o and *H*e values ranged from 0.40 to 0.88 (mean=0.55) and 0.58 to 0.81 (mean=0.69), respectively, and the *PIC* ranged from 0.519 to 0.754 (mean=0.625) for each locus (Table 3). Primer information of the six markers is shown in Supplementary Table S4.

**Table 3:** Number, length, frequency, density, and GC content of perfect STRs

Locus	*N*a	*N*	*H*o	*H*e	*PIC*
JR05	4	16	0.875	0.659	0.590
JR09	7	16	0.750	0.808	0.754
JR10	6	15	0.400	0.577	0.524
JR12	6	15	0.400	0.811	0.751
JR18	5	15	0.467	0.602	0.519
JR20	4	15	0.400	0.701	0.613

*N*a, observed number of alleles; *N*, sample number; *H*o, mean observed heterozygosity; *H*e, mean expected heterozygosity; *PIC*, polymorphic information content.

### Variation and genetic relatedness analysis based on genotyping data of STRs

**Figure 4:**
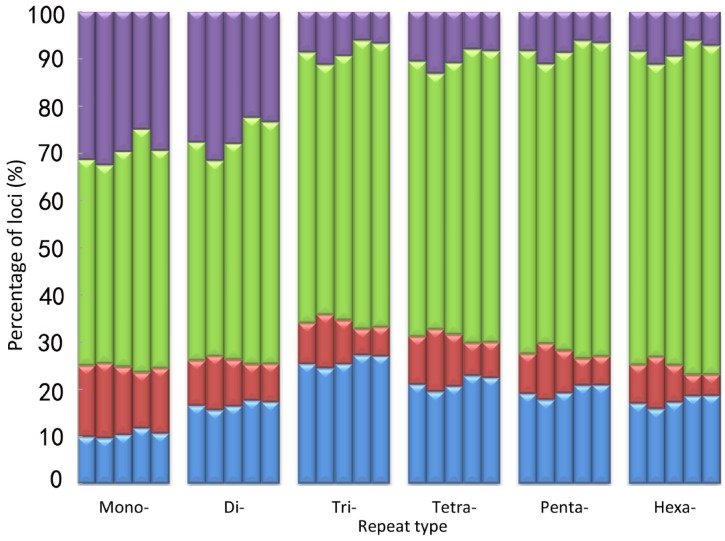
Comparison of the four allelotype categories for each repeat type among the five Tibetan macaques

**Figure 5:**
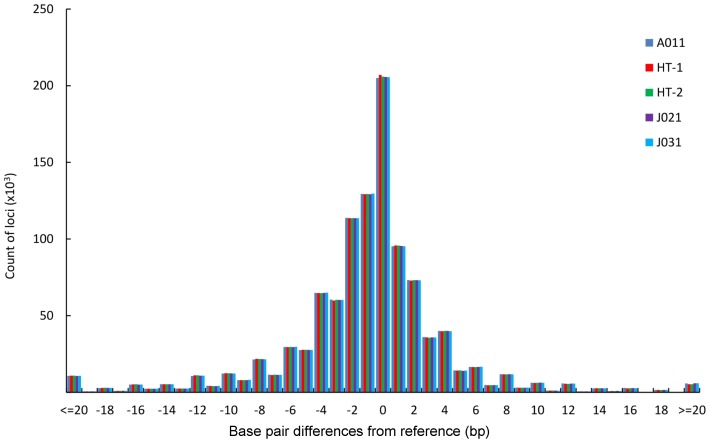
Distribution of allele size differences in STRs from the reference for the five *M. thibetana* individuals

To obtain more accurate and reliable results, we focused on the 526 699 loci called in all five macaques, including 354 577 mono-, 96 370 di-, 20 271 tri-, 41 166 tetra-, 12 042 penta-, and 2 273 hexa-nucleotide loci. We divided these loci into four allelotype categories: homozygous reference where both alleles are aligned to the reference; heterozygous reference where one allele is aligned to the reference; homozygous nonreference where both alleles are not mapped to the reference but are the same; and heterozygous nonreference where both alleles are different and do not match the reference. For mononucleotide repeats, as shown in Figure 4 and Supplementary Table S5, the proportion of homozygous reference/nonreference loci was lower in SC than in HS, whereas, the proportion of heterozygous reference/nonreference was higher in SC than in HS. The distributional pattern of the four allelotype categories in the other repeat types was similar to that in the mononucleotide repeats (Figure 4 and Supplementary Table S5). These results showed that Tibetan macaques in HS had a higher proportion of homozygous reference and nonreference loci than that in SC for each repeat type. Conversely, SC individuals had more heterozygous reference/nonreference loci (Figure 4). Overall, there was a far higher number of homozygous STRs than heterozygous STRs in all five Tibetan macaques, and the difference was significant (*P*<0.05, *t*-test). The percentage of homozygous loci was positively correlated with the length of the repeat motif (Figure 4). Compared with the reference alleles, the proportion of base pair deletions in the STRs was greater than that of insertions for the Tibetan macaques (*P*<0.05, *t*-test), and the proportion of insertions and mean variations in STR allele size were slightly larger in SC than in HS (Supplementary Table S6). On average, these loci demonstrated a 3.4 bp difference, with differences greater than 5 bp accounting for approximately 18.30% in each individual. Furthermore, insertions accounted for 30.60% of loci and base pair deletions accounted for 49.83% of loci (Figure 5 and Supplementary Table S6).

To measure genetic relatedness, 41 166 tetranucleotide STR loci called in all five macaques were used for relatedness analysis. The phylogenetic tree constructed by neighbor-joining based on genotyping data of the tetranucleotide loci showed that the three individuals from SC were clustered onto one branch and the two individuals from HS were clustered onto another branch. On the SC branch, the two individuals from Jianyang and one individual from EM showed little genetic differentiation (Supplementary Figure S2). This result was consistent with their geographical distribution.

(Blue) homozygous reference; (red) heterozygous reference; (green) homozygous nonreference; (purple) heterozygous nonreference. Columns represent the proportion of the four allelotype categories of A011, J021, J031, HT-1, and HT-2 from left to right for each repeat type, respectively.

## DISCUSSION

### Genome assembly and STR distribution of *M. thibetana*

Recently, with the development of next-generation sequencing technology and advancement in bioinformatics analysis, considerable research on genetic variation in primates has been conducted based on genome-wide sequencing, including on humans (1000 Genomes Project Consortium et al., 2015), rhesus macaques (Liu et al., 2017b; Xue et al., 2016; Zhong et al., 2016), and cynomolgus macaques (Osada et al., 2015). To date, however, few genome-scale studies have been reported in Tibetan macaques, except for the previous identification of 11.9 million single nucleotide variants (SNVs) (Fan et al., 2014). Sequencing technology now offers an unprecedented opportunity to examine STR loci of good quality and STR variations among Tibetan macaques. In the present study, based on resequencing data, STR variations and characterizations were investigated at the genome-level in Tibetan macaques. These data provide a novel genomic resource for *M. thibetana* species and enrich the database on genetic variants in macaques.

The identification and development of Tibetan macaque STR molecular markers reported in previous studies were based on the construction of genomic libraries (Jia et al., 2011, 2012; Li et al., 2014), which is both labor intensive and time consuming. Here, we employed resequencing data generated on the Illumina platform to characterize the distributional pattern of STRs. Analysis of the genome showed an average GC content of 40.48%, similar to that reported in other macaque genomes (Zimin et al., 2014). The N50 and mean length of the assembled genome were 4 966 bp and 2 172.38 bp, respectively, sufficient for detecting STR loci. We found that the proportion of STRs in the assembled genome was slightly lower than the 0.83%–0.88% obtained in other macaques (Liu et al., 2017b). This may be because short contigs of less than 150 bases were deleted from the assembly to obtain more reliable and accurate STR loci, leading to an incomplete genome. Mononucleotide STR loci are the most abundant repeat type in most organisms (Sharma et al., 2007), as was also found in our study. Dinucleotide and tetranucleotide STRs were the second most abundant STRs and showed similar frequencies, which is in accordance with that observed in other macaques (Liu et al., 2017b). Furthermore, analysis of GC content and repeat times for each repeat type also showed consistent results with previous study (Liu et al., 2017b). These similarities are a good explanation for the relatively high success rate of cross amplification among macaques (Engelhardt et al., 2017). We discovered that the predominant STR loci contained more A or T bases, except for dinucleotide repeats in which the AC motif was the most abundant, which has also been observed in humans (Subramanian et al., 2003). This could be due to a reported tendency that the poly(A) stretches in the genome might be more biased toward AT base pairs during STR evolution and GC-rich genome sequences are more easily mutated to produce A-rich repeats than AT-rich regions (International Human Genome Sequencing Consortium, 2001; Subramanian et al., 2003).

### Characterization and polymorphism of STRs based on genotyping data

Within the 840 356 potential STRs identified, 62.68% of loci were called in all five individuals and 4.20% of loci were only present in one individual. This may be due to the low sequencing coverage, besides true differences among different individuals. Notably, however, STRs mapped to the chromosomes exhibited similar distributional regularity with the distribution of STRs observed in other macaque chromosomes (Liu et al., 2017b). For instance, most STR loci were aligned to chr 1. Several studies have indicated a positive correlation between chromosome size and STR number (Liu et al., 2017b; Qi et al., 2015). Thus, the distribution of chromosome size in *M. thibetana* may be similar to that of the rhesus macaque. In addition, we noted that GC-rich STR motifs showed lower polymorphism, which may be due to the correlation between GC-rich sequences and functionality (Benjamini & Speed, 2012).

Analysis of allele number of STR loci showed that only 1.41% of the loci showed no variation among the five individuals. A similar occurrence has been reported in cattle genome research (Xu et al., 2016). Here, loci with only one allele were found at highest proportion among trinucleotide STRs compared with other STRs. Furthermore, pSTRs showed the lowest proportion among tri- and hexa-nucleotide STRs. These two results showed that variation in tri- and hexa-nucleotide STRs was smaller than that in the other four STR types. This could be attributed to the fact that these two STR types are more frequent in exons (Qi et al., 2016), which are related to gene function (La Spada et al., 1991; MacDonald et al., 1993). However, pSTRs were overrepresented in general, indicating that high polymorphic STRs were relatively abundant in the Tibetan macaque genome. These loci were found at the highest proportion in mono- and di-nucleotide STRs, followed by tetra-nucleotide STRs; however, mono- and di-nucleotide STRs are unstable and more easily produce errors in experiments (Morin et al., 2001; Taberlet et al., 1999). We also found the number of tetranucleotide STRs was higher than that of tri-, penta-, and hexa-nucleotide STRs. Thus, it is more efficient to screen polymorphic loci from tetranucleotide STRs.

From the 3 325 polymorphic tetranucleotide STRs (≥4 alleles) with allele frequencies over 0.1 detected in this study, six loci were used for genetic diversity analysis of the 16 *M. thibetana* samples. The allele number, *H*o, *H*e, and *PIC* results revealed high genetic diversity within Tibetan macaques. Recent study reported 12 polymorphic loci in *M. thibetana* screened from 30 STR sites in humans (Yang et al., 2017), though the amplification efficiency was lower than these STR loci identified in our study. This result indicated that the polymorphic STR loci identified in the present study can be used for amplification in the *M. thibetana* genome and polymorphisms of STRs were relatively high. The combination of STR loci searched in the assembled genome and polymorphic STRs identified in this study for isolation of STRs was both efficient and time-conserving.

### STR variation among five Tibetan macaque individuals

Based on the 526 699 STR loci called in all five samples, we found Tibetan macaques in SC had more heterozygous reference/nonreference loci than those in HS. This indicated that genetic diversity in Tibetan macaques was higher in SC than in HS. Previous studies based on mitochondrial DNA suggest that overall genetic diversity in *M. thibetana* is high, and that genetic variation is higher in the SC population than in the HS population (Li et al., 2008; Zhong et al., 2013). Our results are consistent with these conclusions. Analysis of SNV distributions in the Tibetan macaque revealed that there was a higher number of homozygous SNVs than heterozygous SNVs (Fan et al., 2014). A similar occurrence was also found in the distribution of STRs, namely the majority of STRs were homozygous loci in the Tibetan macaque (*P*<0.05, *t*-test). Compared with the rhesus macaque reference alleles, the proportion of base pair deletions in the Tibetan macaque was greater than that of insertions (*P*<0.05, *t*-test), consistent with the small indels genotyped with the Genome Analysis Toolkit (GATK) (Fan et al., 2014). The average length of loci was 19.19 bp in the present assembly, which is slightly shorter than the 20.14 bp for *M. mulatta* (Liu et al., 2017b). These results indicate that a large proportion of STR loci may have longer repeats in rhesus macaques than orthologous loci in *M. thibetana*. This has also been reported from human-chimpanzee genomic sequence alignments (Webster et al., 2002). The STR loci in each Tibetan macaque showed an average difference of 3.40 bp from the rhesus macaque. The proportion of insertions and mean variations in STR allele size were slightly larger in the Emei and Jianyang individuals than in the Huangshan individuals, which may reveal differences in allele size of STRs between the two populations. Further studies are needed to examine the effect of these potential STR variations.

### Genetic relationship analyses of microsatellites

The neighbor-joining tree constructed based on tetranucleotide STRs classified the five individuals into two different branches according to their geographic origin. Little genetic differentiation was observed between the Jianyang and Emei individuals. Tibetan macaques from Sichuan and Huangshan were classified into two different subspecies (*M. thibetana thibetana* and *M. thibetana huangshanensis*) based on their external morphological and anatomical variations (Jiang et al., 1996), and the neighbor-joining tree confirmed the genetic differentiation between the two populations. Previous study based on mitochondrial DNA has demonstrated that the two populations exhibit significant genetic differentiation (Zhong et al., 2013), which may be the result of geographical barriers. Specifically, gene flow between the two populations is obstructed, leading to genetic differentiation (Zhong et al., 2013). Owing to the limited number of samples, our results provide an initial understanding of the genetic variation of STRs in Tibetan macaques, and future studies are needed to investigate population genetic variations using more samples from distinct populations.

## CONCLUSIONS

In summary, we performed STR characterization in the Tibetan macaque genome and provided a genome-wide atlas of microsatellite distribution with next-generation sequencing data. The polymorphic STR loci identified from the reference genome exhibited good amplification efficiency and can be well used for population genetics. This profiling will be conducive for future genome-wide genetic analyses of the Tibetan macaque at the population scale. Analysis of genome-wide STRs also preliminarily revealed variation in the genetic diversity among Tibetan macaque populations. This study contributes to our further recognition of the genetic variation among Tibetan macaque species.
